# Supplementary material for: HIV-1 Circulating Recombinant Forms (CRFs) and Unique Recombinant Forms (URFs) in Israel, 2010–2018
Source: Viruses. 2022 Sep 6;14(9):1970. doi: 10.3390/v14091970 (PMC9502407; doi:10.3390/v14091970)
Supplement: Supplementary file 1 [file viruses-14-01970-s001.zip › viruses-1835931-supplementary/Supplementary Materials_OM figs1,s2 table S1 excel and s2 FINAL 070722.pdf]

Supplementary Materials

Table S2- Detailed list of all 213 cases of HIV-1 recombinants. Sample numbers of all 213 cases, results of RIP and of RDP5 (using PR and RT sequences either separately or combined PR RT sequences) is shown for each case. Column 1(from the left) presents the accession numbers of sequences if available. Column 2 shows the number of samples. Column 3 depicts the results of the Recombinant Identification Programme (RIP) analysis in the protease (PR) region. Column 4 shows the RIP analysis in the reverse transcriptase region (RT). Column 5 represents the results of RIP analysis in both PR and RT regions. Column 6 shows the results of Recombinant Detection Programme version 5.5 (RDP5) in the RT region. Column 7 represents the results of RDP5 in both PR and RT regions. Column 8 shows the conclusion of HIV-1 subtyping. Column 9 presents the comments, which are related to HIV-1 subtyping results. In cases with discordant results (15/213), the phylogenetic analysis prevailed. In such a case the comment " Final determination of HIV-1 subtyping by phylogeny" is mentioned in the comments. N/A- Non-Available (file in excel format).

Table S3: Proportion of TDRM and HIVdrm in recombinants in newly diagnosed people living with HIV-1 (PLHIV) in Israel 2010-2018.

			HIV-1 Subtype (N)																				
**Drug class**	**TDRM**	**All N= 213**	**AG 65**	**AE 36**	**AE/B 3**	**AG/ AE/ A3 23**	**B/F1 15**	**AG/ A4, 14**	**A6/B 8**	**C/A1 6**	**AG/B 6**	**AG/ A6 3**	**AG/G 6**	**B/C 4**	**B/D 5**	**Minor subty pes 19**	**AG vs AG/A4**	**AG vs Minor subtypes**	**AG vs AG/AE/ A3**	**AG vs B/F1**	**AG vs AG/G**	**AG/A4 vs AG/B**	**AG/A4 vs Minor subtypes**
**PI, n (%)**	L10F	**3 (1.4)**	**1 (1.5)**													***2 (10.5)***		*0.063*					
	K20T	**1 (0.5)**		**1 (2.8)**																			
	**D30N**	**1 (0.5)**				**1 (4.3)**																	
	L33F	**1 (0.5)**			**1(33.3)**																		
	K43T	**1 (0.5)**														***1 (5.3)***							
	**M46L**	**2 (0.9)**	**1 (1.5)**			**1 (4.3)**													*0.437*				
	**V82MS**	**1 (0.5)**														***1 (5.3)***							
	**L90M**	**1 (0.5)**													**1 (20)**								
**NRTI, n (%)**	**L74V**	**1 (0.5)**													**1 (20)**								
	**T215**A**D** N	**1 (0.5)**											**1 (16.6)**										
	**K219R**	**1 (0.5)**							**1 (12.5)**														
**NNRTI, n (%)**	**K103NS**	**6 (2.8)**	**2 (3.1)**				**2(13.3)**						**1 (16.6)**			***1 (5.3)***				*0.105*	*0.119*		
	**K103N**	**5 (2.3)**	**2 (3.1)**				**2(13.3)**									***1 (5.3)***				*0.105*			
	**K103S**	**1 (0.5)**											**1 (16.6)**										
	E138AG KQ	**20 (9.4)**	**3 (4.6)**					**14 (100)**			**1 (16.7)**		**1 (16.6)**			***1 (5.3)***	***<0.001***				*0.225*	***<0.001***	***<0.001***
	E138A	**18 (8.5)**	**2 (3.1)**					**14 (100)**			**1 (16.7)**					***1 (5.3)***	***<0.001***					***<0.001***	***<0.001***
	E138G	**1 (0.5)**											**1 (16.6)**										
	E138Q	**1 (0.5)**	**1 (1.5)**																				
	V179E	**4 (1.9)**	**3 (4.6)**					**1 (7.1)**									*0.700*						
	**G190A**	**1 (0.5)**											**1 (16.6)**										

TDRM, transmitted drug resistance mutations, highlighted in bold blue; HIVdrm- HIV-1 drug resistance mutations; NRTI, nucleoside reverse transcriptase inhibitors; NNRTI, non-nucleoside transcriptase inhibitors; PI, protease inhibitors. Data are presented as n (%). Differences in proportions were measured using the chi-squared test. Empty cells, n=zero.

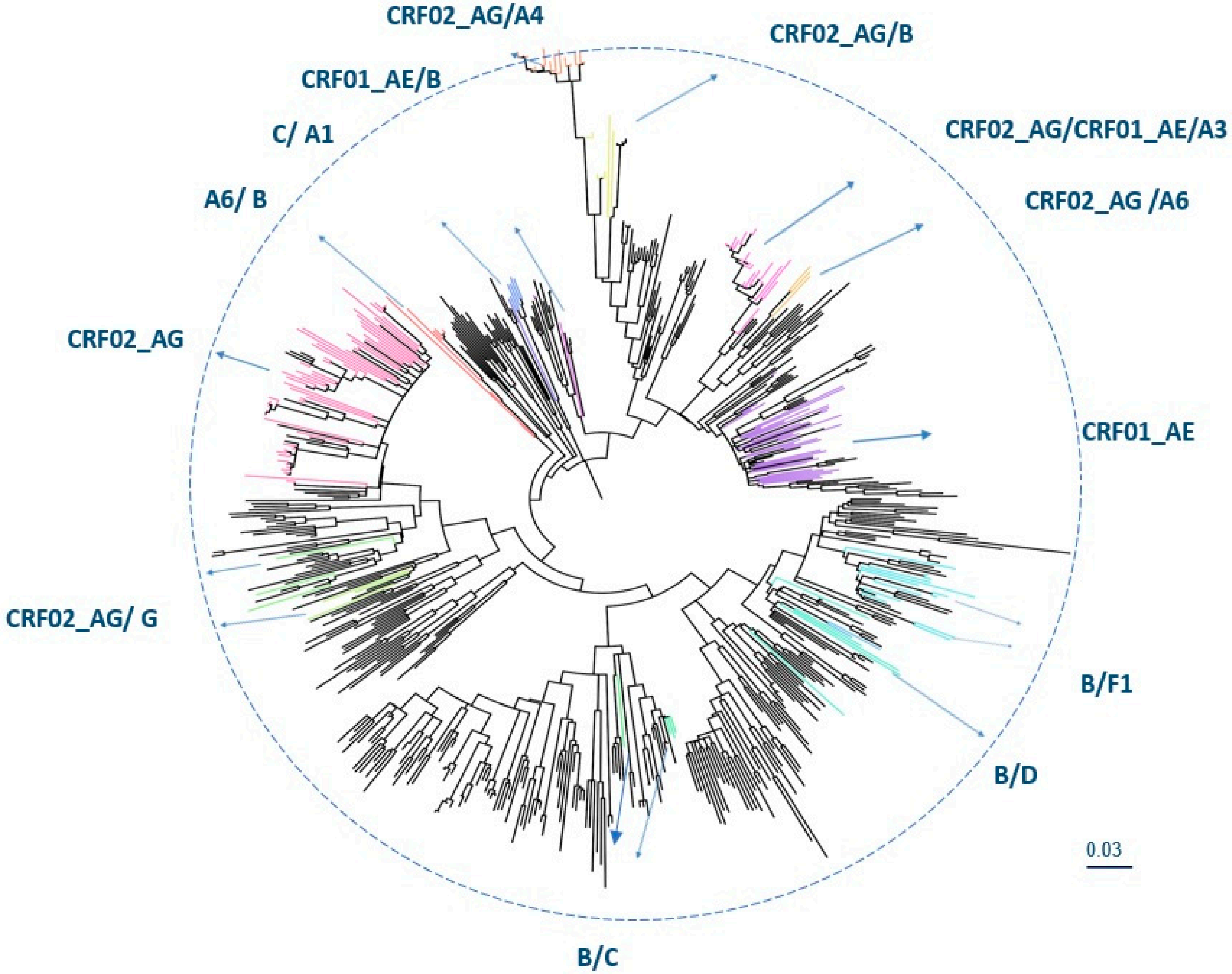
CRF02_AG/A4
CRF02_AG/B
CRF01_AE/B
C/ A1
CRF02_AG/CRF01_AE/A3
A6/ B
CRF02_AG /A6
CRF02_AG
CRF01_AE
CRF02_AG/ G
B/F1
B/D
0.03
B/C

Figure S1: **Phylogenetic analysis of HIV-1 recombinants in Israel, 2010-2018.** Phylogenetic tree of the *pol* region of HIV-1 from 213 patients was constructed using the maximum likelihood (ML) with GTR + G+I model. Included were also 410 reference sequences (pure and recombinant forms) obtained from the Los Alamos database. The tree was visualized in Fig Tree version 1.4.4. HIV-1 recombinants are colored as follows: CRF02_AG in pink, CRF01_AE in purple, CRF01_AE/B in violet, CRF02_AG/CRF01_AE/A3 in rose, B/F1 in turquoise, CRF02_AG/A4 in brown, A6/B in red, C/A1 in blue, CRF02_AG/B in yellow, CRF02_AG/A6 in gold, CRF02_AG/G in green, B/C in Caribbean green, B/D in magic mint. Reference sequences and cases of < 3 sequences that did form a cluster and were considered minor inter-subtype (N=19) are presented in black.

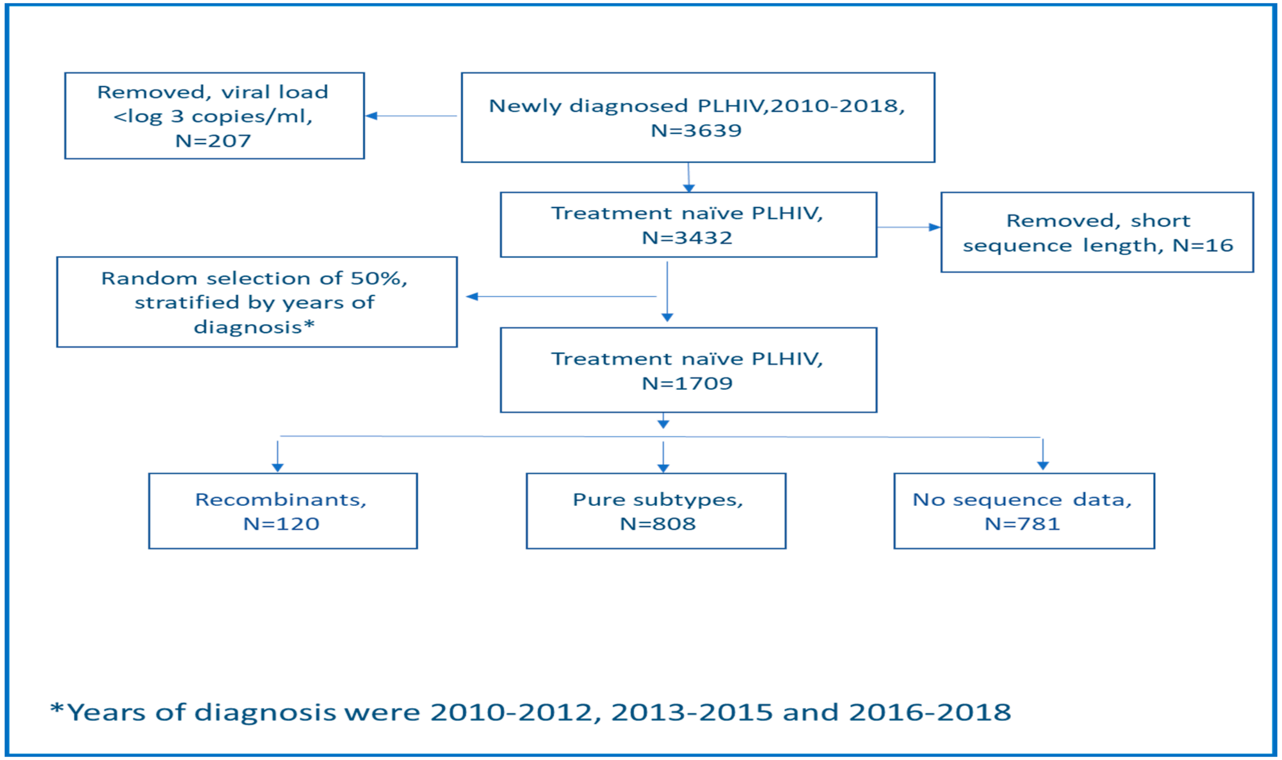

Figure S2: Flow diagram of a random selection of representative sample (50%) from all treatment naïve HIV-1 positives diagnosed in 2010-2018. All patients diagnosed in 2010-2018 were assessed. Patients with HIV-1 viral load <3 log c/ml at diagnosis were excluded. Sequences with short lengths (<918 nucleotides of PR and RT combined) were also excluded. For random selection stratification to three groups of years of diagnosis (2010-2012, 2013-2015, 2016-2018) was performed.
